# Supplementary material for: HER2-CD3-Fc Bispecific Antibody-Encoding mRNA Delivered by Lipid Nanoparticles Suppresses HER2-Positive Tumor Growth
Source: Vaccines (Basel). 2024 Jul 21;12(7):808. doi: 10.3390/vaccines12070808 (PMC11281407; doi:10.3390/vaccines12070808)
Supplement: Supplementary file 1 [file vaccines-12-00808-s001.zip › vaccines-3060522-supplementary.pdf]

Supplementary materials for

Lipid Nanoparticle-Mediated HER2-CD3-Fc Bispecific Antibody mRNA Delivery Potently Suppresses HER2-Positive Tumor Growth

Liang Hu[1], Shiming Zhang[1], John Sienkiewicz[1], Hua Zhou[1], Robert Berahovich[1], Jinying Sun[1], Michael Li[1], Adrian Ocampo[1], Xianghong Liu[1], Yanwei Huang[1], Hizkia Harto[1], Shirley Xu[1], Vita Golubovskaya[1*], Lijun Wu[1,2*]

[1] Promab Biotechnologies, 2600 Hilltop Drive, Richmond, CA 94806, USA;

[2] Forevertek Biotechnology, Janshan Road, Changsha Hi-Tech Industrial Development Zone, Changsha 410205, China;

* Correspondence: vita.gol@promab.com (V.G.); john@promab.com (L.W.); Tel.: +1-510-974-0697 (V.G.); +1-510-529-3021 (L.W.)

The file includes the following data:

Figure S1. Amino acid sequence of the HER2-CD3-Fc bsAb

Figure S2. Expression of the HER2-CD3-Fc bsAb in cancer cells

Figure S3. Functional analysis of the HER2-CD3-Fc mRNA-LNPs using RTCA

Figure S4. The HER2-CD3-Fc mRNA-LNPs induce T cells to secrete effector molecules and cytokines against HER2-positive target cells

Figure S5. The HER2-CD3-Fc bsAb mediates a highly specific antitumor effect dependent on HER2

Figure S6. The HER2-CD3-Fc mRNA-LNPs mediate a highly specific antitumor effect dependent on HER2

METDTLLLWVLLLWVPGSTGAASDIQMTQSPSSLSASVGDRVTITCRASQDVNTAVAWY
QQKPGKAPKLLIYSASFLESGVPSRFSGSRSGTDFTLTISSLQPEDFATYYCQQHYTTPPTFG
QGTKVEIKGGGGSGGGGSGGGGSEVQLVESGGGLVQPGGSLRLSCAASGFNIKDTYIHWVR
QAPGKGLEWVARIYPTNGYTRYADSVKGRFTISADTSKNTAYLQMNSLRAEDTAVYYCSR
WGGDGFYAMDVWGQGTLVTVSSGGGGSGGGGSGGGGSEVQLLESGGGLVQPGGSLRLSCA
ASGFTFSTYAMNWVRQAPGKGLEWVSRIRSKYNNYATYYADSVKGRFTISRDDSKNTLYL
QMNSLRAEDTAVYYCVRHGNFGNSYVSWFAYWGQGTLVTVSSGGGGSGGGGSGGGGSQA
VVTQEPSLTVSPGGTVTLTCGSSTGAVTTSNYANWVQEKPGQAFRGLIGGTNKRAPGTPA
RFSGSLLGGKAALTLSGAQPEDEAEYYCALWYSNLWVFGGGTKLTVLSRENLYFQGTHTC
PPCPAPEAAGGPSVFLFPPKPKDTLMISRTPEVTCVVVDVSHEDPEVKFNWYVDGVEVHN
AKTKPREEQYNSTYRVVSVLTVLHQDWLNGKEYKCKVSNKALGAPIEKTISKAKGQPREP
QVYTLPPSRDELTKNQVSLTCLVKGFYPSDIAVEWESNGQPENNYKTTPPVLDSDGSFFLY
SKLTVDKSRWQQGNVFSCSVMHEALHNHYTQKSLSLSPGK

Figure S1. Amino acid sequence of the HER2-CD3-Fc bsAb.

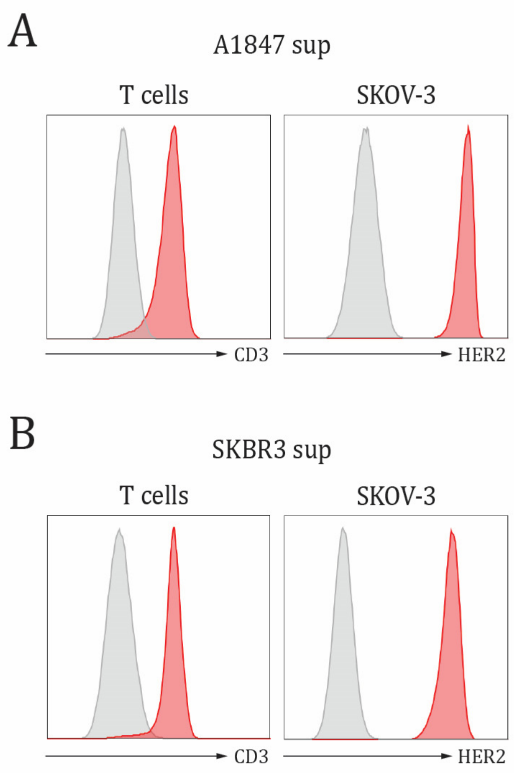

Figure S2. Expression of the HER2-CD3-Fc bsAb in cancer cells. A1847 (**A**) or SKBR3 (**B**) cells were transfected with 20 µl LNPs containing 1 µg of the bsAb mRNA, and the culture medium was collected 72 h later. The medium was used at a dilution of 1:10 to stain human T cells and SKOV-3 cells (red histograms). Non-transfected A1847 or SKBR3 cell medium was used at 1:10 as a negative control (grey histograms). The secondary antibody was APC anti-human Fc.

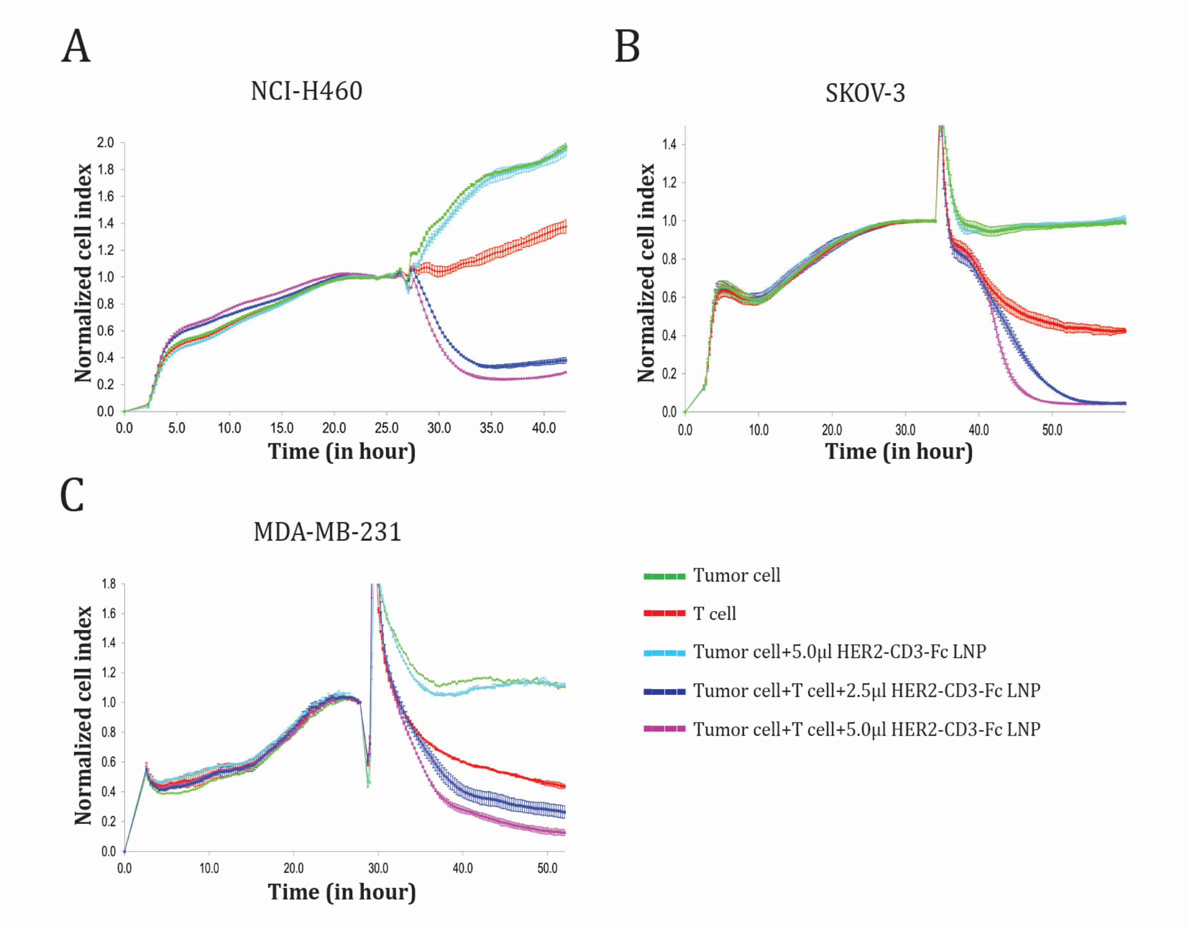

Figure S3. Functional analysis of the HER2-CD3-Fc mRNA-LNPs using RTCA. The target cells NCI-H460 (**A**), SKOV-3 (**B**), or MDA-MB-231 (**C**) were seeded in triplicate in a 96-well E-plate overnight. The next day, T cells and different amounts of the HER2-CD3-Fc mRNA-LNPs were added to the target cells. The ratio of the T cells to the target tumor cells was 10:1. The impedance of the target cell monolayer was monitored by the RTCA system. Mean ± SD impedance is plotted and normalized to the time of T cell addition.

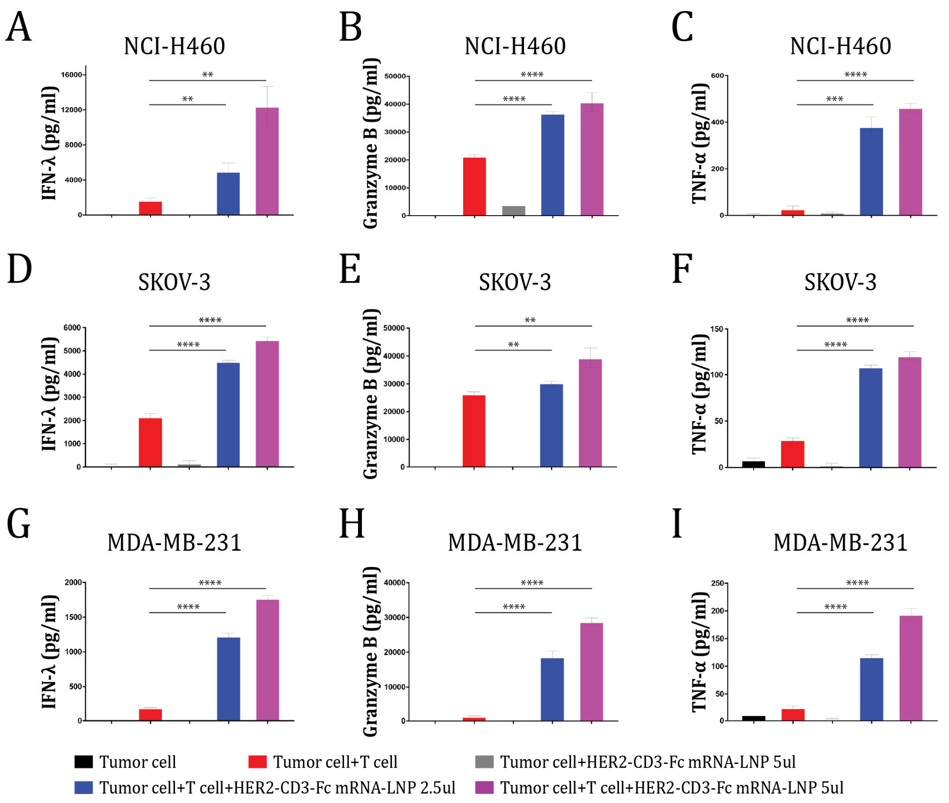

Figure S4. The HER2-CD3-Fc mRNA-LNPs induce T cells to secrete effector molecules and cytokines against HER2-positive target cells. The target cells NCI-H460 (**A-C**), SKOV-3 (**D-F**), or MDA-MB-231 (**G-I**) were cultured in triplicate with T cells and different amounts of the HER2-CD3-Fc mRNA-LNPs. After 24 h, the culture medium was collected and analyzed by ELISA for IFN-λ (A,D,G), granzyme B (B,E,H) and TNF-α (C,F,I) levels. Data are representative of at least two independent experiments (three replicates per group). Data are presented as the means ± SD. A p value < 0.05 was considered statistically significant (** $p < 0.01$, *** $p < 0.001$, and **** $p < 0.0001$).

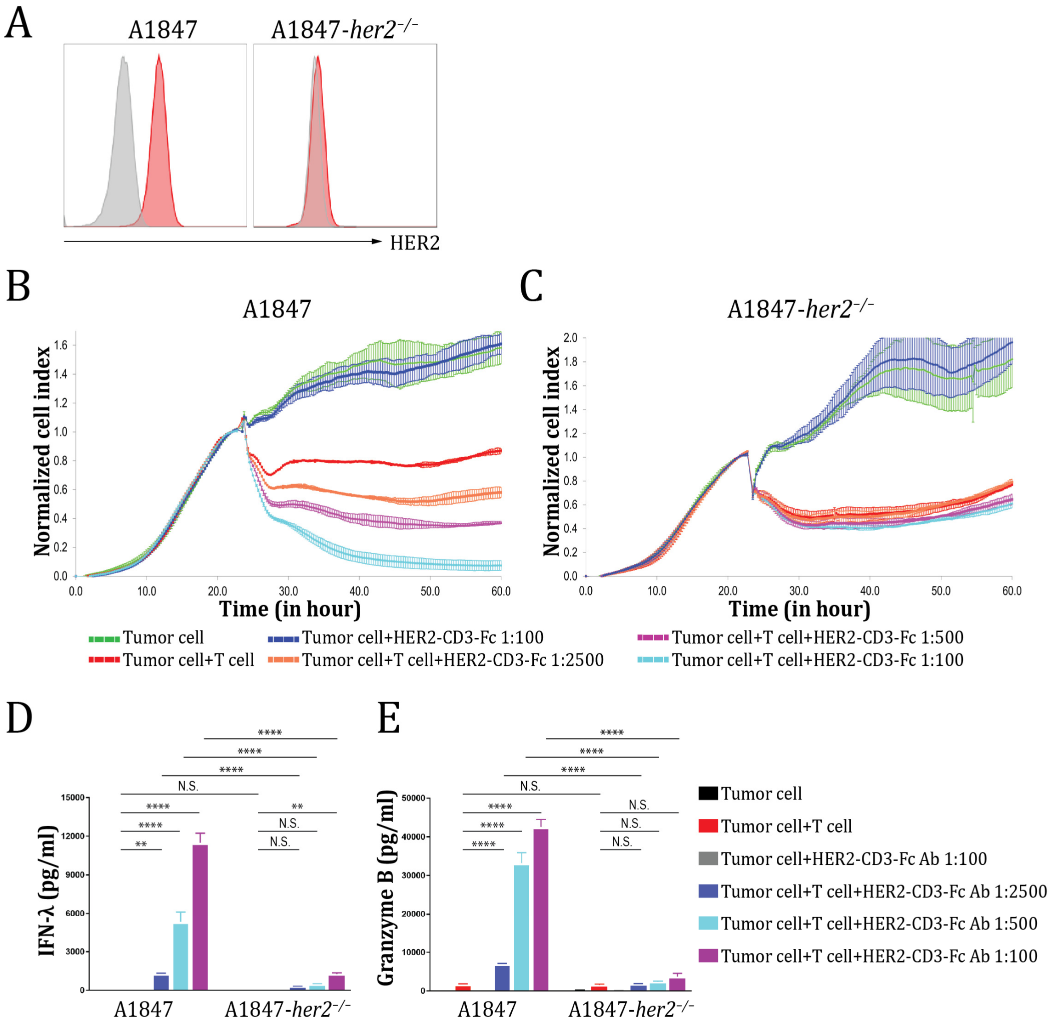

Figure S5. The HER2-CD3-Fc bsAb mediates a highly specific antitumor effect dependent on HER2. (**A**) Analysis of A1847 *her2* knockout cells. The *her2* knockout cells were generated using the CRISPR/Cas9 system, and the lack of HER2 expression was verified by flow cytometry using 1:10-diluted bsAb-containing HEK293S cell medium (red histograms) or control HEK293S medium (grey histograms), followed by APC anti-human Fc secondary Ab. (**B-E**) Analysis of HER2-CD3-Fc bsAb-mediated T cell cytotoxicity against A1847 *her2* knockout cells. A1847 (B) or A1847-*her2*$^{-/-}$ (C) cells were seeded in triplicate in a 96-well E-plate overnight. The next day, T cells and bsAb-containing HEK293S cell medium was added to the tumor cells. The ratio of the T cells to the target tumor cells was 10:1. The impedance of the target cell monolayer was monitored by the RTCA system. Mean ± SD impedance is plotted and normalized to the time of T cell addition. The culture medium was collected and analyzed by ELISA for IFN-λ (D) and granzyme B (E) levels. Data in (B-E) are representative of at least two independent experiments (three replicates per group). Data in (D-E) are presented as the means ± SD. A p value < 0.05 was considered statistically significant (** $p < 0.01$, and **** $p < 0.0001$). A p value ≥ 0.05 was statistically not significant (N.S.).

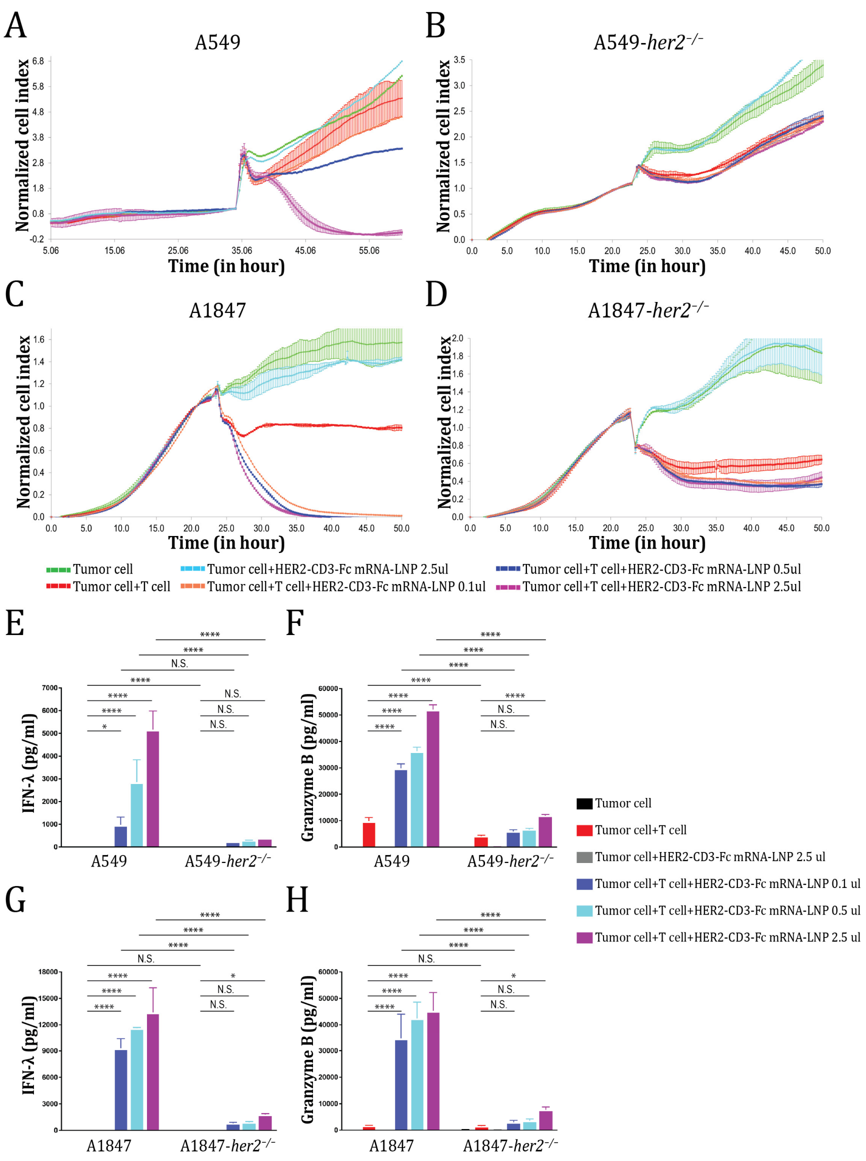

Figure S6. The HER2-CD3-Fc mRNA-LNPs mediate a highly specific antitumor effect dependent on HER2. The target cells A549 (**A**), A549-*her2*$^{-/-}$ (**B**), A1847 (**C**), or A1847-*her2*$^{-/-}$ (**D**) were seeded in triplicate in a 96-well E-plate overnight. The next day, T cells and HER2-CD3-Fc mRNA-LNPs were added to the tumor cells. The ratio of the T cells to the target tumor cells was 10:1. The impedance of the target cell monolayer was monitored by the RTCA system. Mean ± SD impedance is plotted and normalized to the time of T cell addition. The culture medium was collected and analyzed by ELISA for IFN-λ (E,G) and granzyme B (F,H) levels. Data are representative of at least two independent experiments (two replicates per group). Data in (E-H) are presented as the means ± SD. A p value < 0.05 was considered statistically significant (* $p < 0.05$, and **** $p < 0.0001$). A p value ≥ 0.05 was statistically not significant (N.S.).
